# More Than One Enzyme: Exploring Alternative FMN-Dependent L-Lactate Oxidases for Biosensor Development

**DOI:** 10.1021/acsomega.4c01897

**Published:** 2024-06-26

**Authors:** Lidiia Tsvik, Shulin Zhang, Danny O’Hare, Dietmar Haltrich, Leander Sützl

**Affiliations:** † Laboratory of Food Biotechnology, Department of Food Science and Technology, University of Natural Resources and Life Sciences, Muthgasse 11, Wien, Vienna A-1190, Austria; ‡ Doctoral Programme ‘Biomolecular Technology of Proteins (BioToP)’, University of Natural Resources and Life Sciences, Muthgasse 18, Wien, Vienna A-1190, Austria; § Department of Bioengineering, Imperial College London, London SW72AZ, U.K.

## Abstract

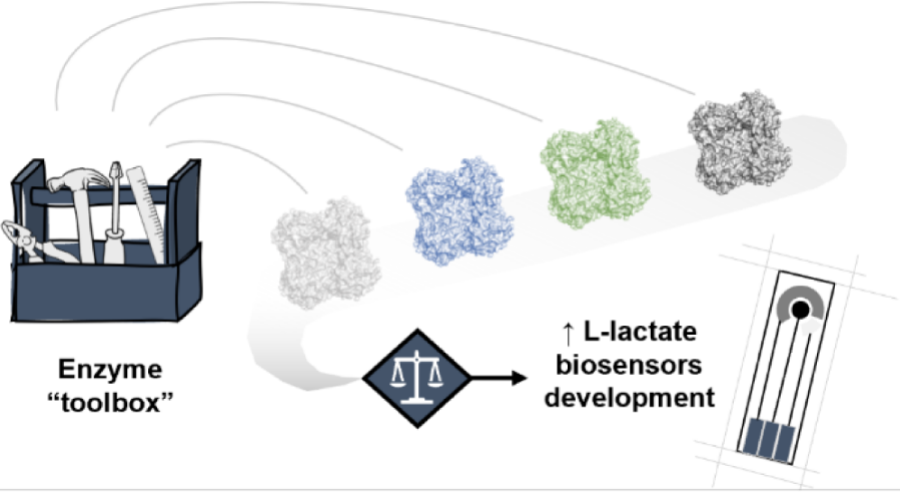

The α-hydroxy acid oxidoreductase (HAOx) family contains a diverse group of enzymes that can be applied in biosensors for L-lactate detection, most prominently lactate oxidase (LOx). The limited availability and a lack of diversity of L-lactate-oxidizing enzymes have currently hindered advancements in L-lactate biosensor development. Until now, the field has mostly relied on a single, commercially available enzyme, namely *Aerococcus viridans* L-lactate oxidase (*Av*LOx). In this study, we present newly discovered alternative L-lactate oxidases that exhibit a narrow substrate specificity and varied kinetic efficiencies toward L-lactate, making them suitable for integration into existing biosensor configurations. Some of these FMN-dependent L-lactate oxidases could be obtained in substantial amounts from routine *E. coli* expression, potentially facilitating commercial production. Using electrochemical characterization with a mediated biosensor setup, we present 7 enzymes that perform comparable or even better than commercial *Av*LOx. Finally, we show that their electrochemical performance is not directly correlating with their biochemical performance, making predictions of the suitability of enzymes for biosensor applications extremely difficult. Our research emphasizes the significance of expanding the enzyme toolbox of L-lactate oxidases for the development of improved L-lactate biosensors.

## Introduction

Lactic acid, or its conjugate base lactate, is an essential biomarker in medicine or in the food and biopharmaceutical industries.^1−4^ In the food industry, lactic acid concentrations provide insights into fermentation activity, flavor development, as well as overall product stability and microbial contamination.^5^ The biopharmaceutical industry depends on the fast detection of lactic acid to avoid its accumulation in mammalian cell cultures, which can create unfavorable conditions for cell proliferation, viability, and pluripotency.^6^ More specifically, it inhibits the growth of Chinese hamster ovary cells, commonly used for the production of therapeutic proteins.^7−9^ As L-lactate is a metabolic product of anaerobic glycolysis, it also occurs in most human tissues^10^ and monitoring its levels is important in clinical diagnostics, intensive care, and sports medicine.^2,11^ Measuring L-lactate levels provides a comprehensive insight into a patient’s condition after medical treatment or can aid in evaluating athletes’ performances during extensive training.^11^

Recent advancements in digital wellness devices and their integration of biomarker-specific sensors^12^ have led to the development of easy-to-use and pain-free sensors. These devices show promising potential as a viable alternative to conventional invasive methods, where patients’ blood samples are analyzed by spectrophotometric, fluorometric, and colorimetric tests, or more sophisticated methods like HPLC and LC-MS.^1,3^ Such non- or minimal-invasive L-lactate biosensors, measuring L-lactate levels in interstitial fluid, sweat, breath or tears,^13,14^ have the advantage of being much better accepted by patients and due to their convenience, also have great success as lifestyle products.^14^ As a result, they have already been well developed and refined over the past decade, making them some of the most studied sensors in this field.

**Figure 1:**
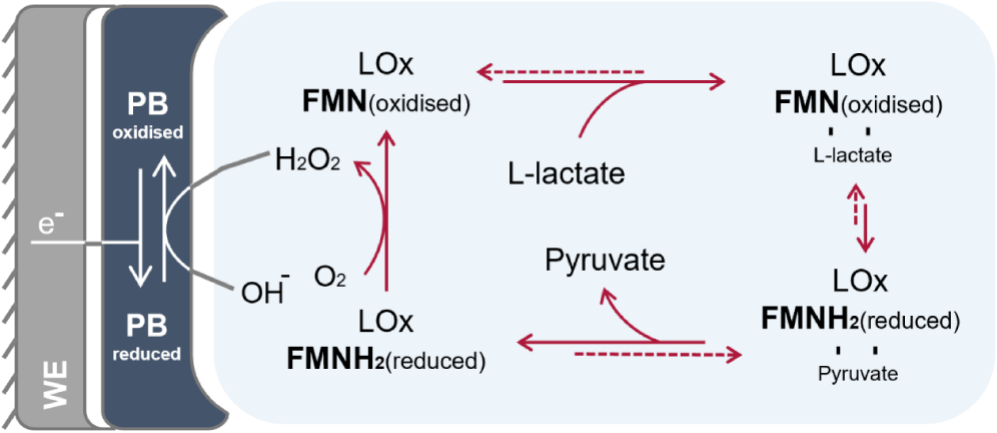
Reaction mechanism of L-lactate utilized in a biosensor in the presence of oxygen (O_2_) as an electron acceptor and PB as a mediator immobilized on a working electrode (WE)^27.^

For most L-lactate biosensors, L-lactate oxidase (LOx) is the preferred biocatalyst. LOx is specific for L-lactate and thus can be applied in biosensors for the detection of L-lactate, even in complex samples containing structurally closely related compounds.^15^ Furthermore, LOx shows good compatibility with various immobilization methods and sensor designs that were developed to enhance the sensitivity and operational stability of the biosensor. LOx has been widely used in various designs of lactate-specific biosensors, aiming at applications in medicine,^16−18^ personal and health care monitoring^19^ or food analysis.^20^ LOx belongs to the family of FMN-dependent α-hydroxy acid oxidoreductases (HAOx; (S)-2-hydroxy-acid: oxygen 2-oxidoreductase; EC 1.1.3.15), which catalyzes the oxidation of α-hydroxy acids to the corresponding α-keto acids via a ping-pong reaction mechanism^21,22^ as depicted in Figure 1. Typically, LOx is a homotetramer with the mass of its monomers ranging from 39 to 44 kDa and each containing a noncovalently but tightly bound FMN prosthetic group.^23^ The isoalloxazine ring of FMN is enveloped by amino acids forming the active site, which is covered by a dynamic lid-loop^23^ that plays an important role in substrate binding and product release.^24,25^ This dynamic lid-loop is the least conserved region when comparing amino acid sequences within the HAOx family.^26^

In its first half-reaction, LOx catalyzes the oxidation of L-lactate to pyruvate while FMN is reduced to FMNH_2_. The ensuing half-reaction reoxidizes the cofactor by reducing molecular oxygen to H_2_O_2_ (Figure 1); alternatively, LOx can also employ other electron acceptors such as 2,6-dichlorophenol indophenol (DCIP), albeit to a varying extent.^26^ H_2_O_2_ is an electroactive species and is formed in amounts stoichiometric to the oxidized L-lactate. It can be reduced directly at an electrode or react with suitable redox mediators to generate electric currents that correlate with L-lactate concentrations in the analytes. Since high potentials are needed for the direct oxidation of H_2_O_2_, such measurements might show unfavorable electrochemical interferences. Therefore, current research on L-lactate biosensors focuses on employing redox mediators that can perform the electrochemical reaction at lower potentials and decrease the unspecific current generation.^17^

One such redox mediator is Prussian Blue (PB), a transition metal-based mediator, which has already been applied multiple times in L-lactate biosensor development.^4,27−29^ Its role is to catalyze the reduction of H_2_O_2_ to H_2_O and its selectivity for H_2_O_2_ over O_2_ was confirmed at an optimized potential.^29^ PB can be reduced to Prussian White (Berlin White) at 0 V (vs Ag/AgCl) or more negative potentials, which then mediates a two-electron reduction of H_2_O_2_ in an electrochemical catalytic mechanism. Typically, PB and LOx are coimmobilized in a conducting polymer deposited on an electrode surface when constructing a sensor.^30^ The use of such polymer membrane or hydrogel layers leads to the overall electrode reaction mechanism being limited by substrate mass-transport in the membrane layer instead of enzymatic turnover. This ensures that losses of enzyme activity will not immediately affect sensor sensitivity (current per unit of substrate), and sensor responses are more independent of the sample matrix. Furthermore, LOx typically shows Michaelis constants around 0.5 mM, but the clinical range of interest is 1–20 mM.^30^ Restricting the substrate flux can therefore enable an extension of the dynamic measurement range.

For characterizing such a sensor design, the use of a rotating disc electrode (RDE) is often advantageous. With the adjustable rotational speed of an RDE, the solution mass transport can be influenced independently of the applied potential.^31^ With this, it is possible to use Koutecký–Levich analysis for different applied potentials to quantify mass transport rate constants and enzyme reaction kinetics on the sensor^32^ and thus test and optimize a biosensor design.

To date, most studies on L-lactate biosensors are centered around one single enzyme, LOx from *Aerococcus viridans* (*Av*LOx), which is commercially available from several manufacturers. When applying only a single enzyme though, one is restricted by its specific properties. Also, the potential of one protein sequence to be engineered for certain applications by mutagenesis is limited. These limitations and the lack of availability of alternative enzymes currently hinder advancements in L-lactate biosensor development, a challenge that has been recently noted also by other researchers in the field.^33^ Therefore, expanding the enzyme toolbox for L-lactate biosensors by studying novel LOx enzymes from different sources on electrodes seems to be an immediate necessity.

Finding novel enzymes for these applications can be done by two main approaches: engineering of known biocatalysts toward desired properties, or tapping into Nature’s vast repertoire of sequence variations. Enzyme engineering had been mainly done with *Av*LOx and addressed aspects such as stability,^34−36^ kinetic properties,^24,25,37^ or reactivity with oxygen.^33,38,39^ Interestingly, finding alternative LOx enzymes that might be advantageous for biosensors has not been widely applied, and if, then only studying a single enzyme.^40−42^ Recently, we investigated the sequence space of the HAOx family with the aim of identifying L-lactate-specific oxidases. Based on a sequence similarity network (SSN) and the association of functional relationships of selected members, the identification of various sequence spaces containing enzymes of different substrate reactivities was possible.^26^ Thus, we showed that true L-lactate oxidases within the HAOx family reside in a single phylogenetic clade that also includes *Av*LOx. This clade is clearly separated from a clade containing sequences for L-lactate-specific oxidoreductases that, however, shown negligibly activity with oxygen while reacting with alternative electron acceptors, the L-lactate dehydrogenase clade.^26^ With this comprehensive understanding of the sequence variations associated with LOx activity, we evaluated a selection of enzymes experimentally. The main objective of the present study was to characterize a variety of hitherto not-described enzymes with L-lactate oxidase activity. Our aim was to investigate these enzymes as potential alternatives t*o Av*LOx, particularly within the field of biosensor development. Also, we hypothesized that the immobilization of these enzymes on the electrode can impact their performance, potentially leading to functionalities not predictable solely from studies in solution.

## Results

### LOx Sequence Selection and Alignment

**Figure 2:**
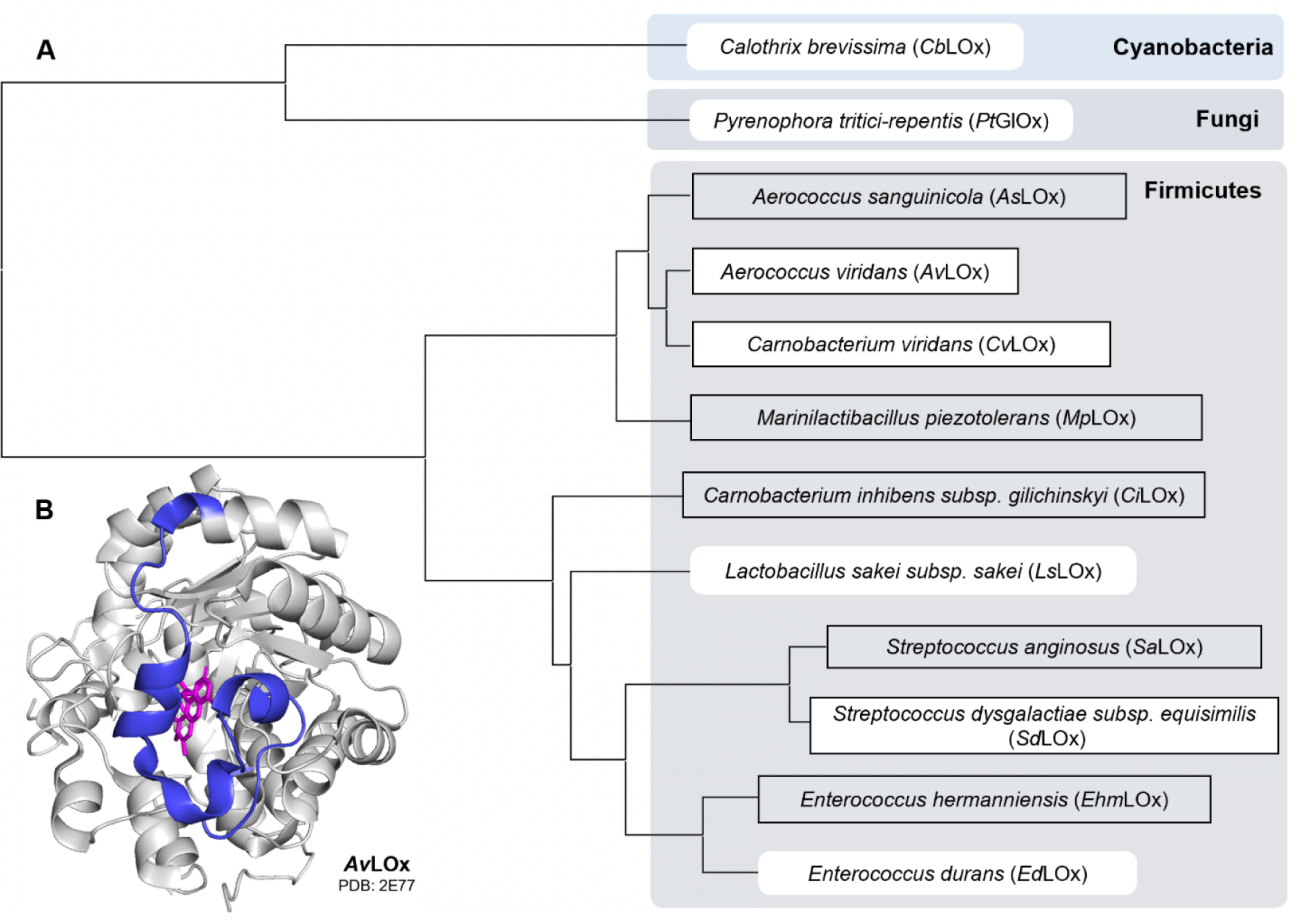
(A) Maximum likelihood phylogenetic tree of the 12 predicted LOx sequences expressed in this study. Sequences that were previously screened for substrate specificity^26^ are indicated with white labels, and sequences that are characterized electrochemically during this study are displayed within black rectangles. (B) *Av*LOx crystal structure^43^ with highlighted lid-loop region (blue) and FMN cofactor (magenta).

Nine bacterial protein sequences from a recently defined LOx clade and two additional sequences that showed good oxidase activity with L-lactate in an initial screening^26^ were selected for recombinant expression together with *Av*LOx as a reference. The full species names and abbreviations of the 12 selected sequences, as well as their phylogenetic relationship, are listed in Figure 2. Corresponding sequence IDs are given in Table S1. The two sequences not belonging to the LOx clade, from the cyanobacterium *C. brevissima* and the fungus *P. tritici-repentis*, are phylogenetically the most distant to the sequences of bacterial (firmicutes) origin, with ∼30% sequence identity to *Av*LOx. Furthermore, a sequence alignment of the 12 proteins (Figure S1) shows that *C. brevissima* and *P. tritici-repentis* have an insertion of 7 and 25 amino acids, respectively, around residues 190–225 in *Av*LOx. These residues are part of the active site lid-loop in LOx and a variation thereof could indicate a variation of substrate preference.

### Expression and Purification

All 12 selected genes were cloned to include an N-terminal polyhistidine tag (His-tag) and an adjacent recognition site for the 3C protease of the human rhinovirus (HRV 3C) for purification. Their expression in *Escherichia coli* was conducted in small-scale shaken flask cultures, followed by a one-step purification using immobilized-metal affinity chromatography (IMAC) as the capture step and HRV 3C digestion to elute the recombinant enzymes without a purification tag. Some of the enzymes could be produced remarkably well with up to 18 mg of pure protein obtained from one gram of wet cell biomass (Table S2). *Av*LOx, on the other hand, was produced only in very low yields and purity. It was therefore replaced by an *Av*LOx preparation from a commercial supplier (Sigma-Aldrich) for all subsequent experiments. The purity of most of the enzyme preparations after a single IMAC step was more than 90%, as judged by the band intensities of the SDS-PAGE analysis (Table S2). The correct incorporation of the FMN cofactor was confirmed by UV–vis absorption spectra, showing typical peaks at around 370 and 450 nm (Figure S2). Purification of *Ls*LOx led to protein precipitation in 11 mM PBS, pH 7.4, upon removal of the purification tag. The protein could be resolubilized with partial restoration of its enzyme activity, when using high salt concentrations, of 500 mM NaCl in the buffer.

### Electron Acceptor and Donor Preferences

In order to confirm that the selected enzymes are indeed specific to L-lactate and oxygen, we performed activity measurements using the electron acceptors, O_2_, DCIP, and ferrocenium ion (Fc^+^ dissociated from FcPF_6_), and the electron donors glycolate, L-lactate, L-2-hydroxyvaleric acid, L-2-hydroxyoctanoic acid, and L-mandelic acid (see Figure S3 for structural comparison). These experiments confirmed that all 11 enzymes are indeed oxidases, showing their most pronounced activity with O_2_, while their dehydrogenase activity with DCIP or Fc^+^ was considerably lower (Table 1). Most of the enzymes are also specific for L-lactate and show negligible activities (≤2%) with other tested α-hydroxy acids. Exceptions were *Sa*LOx and *Sd*LOx, which also showed prominent activity with the larger α-hydroxy acids, L-2-hydroxyvaleric acid, and L-2-hydroxyoctanoic acid. The enzyme from *P. tritici-repentis* generally showed the broadest substrate spectrum with its highest activity on glycolate and was therefore termed a glycolate oxidase (*Pt*GlOx). *Pt*GlOx is the only sequence that was tested from a fungal source. It appeared to be the most distant in the phylogenetic analysis (Figure 2) and showed a long insertion at the active site lid-loop (Figure S1).

**Table 1:**
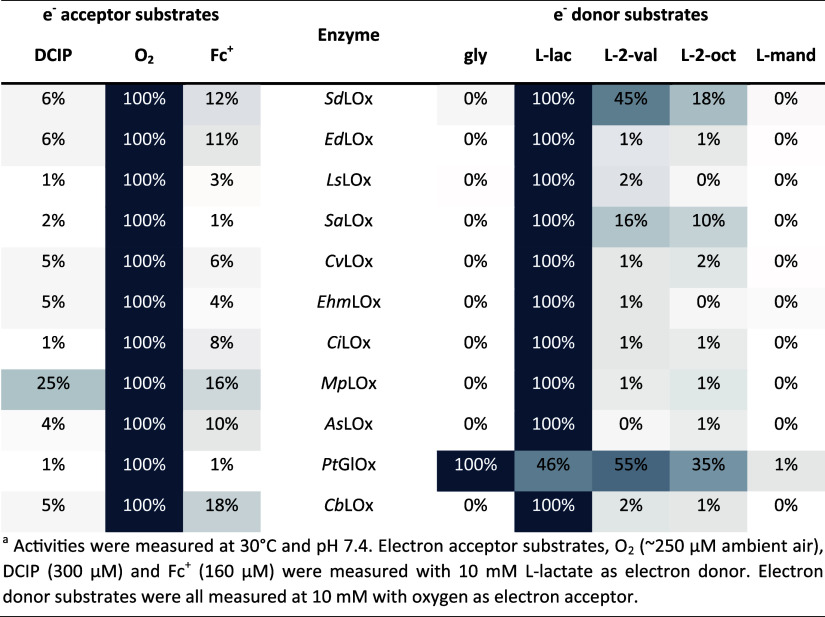
Substrate Specificities of LOxs, Shown as Relative Activities with Different Electron Acceptor and Electron Donor Substrates (Glycolate (gly), L-Lactate (L-lac), L-2-hydroxyvaleric Acid (L-2-val), L-2-hydroxyoctanoic Acid (L-2-oct), and L-mandelic Acid (L-mand))^a^

The enzymes *Ed*LOx, *Cb*LOx, *Ls*LOx, and *Pt*GlOx were not further considered for more detailed biochemical and electrochemical characterization, since they were obtained in low yields and could not be sufficiently purified (*Ed*LOx, *Cb*LOx), were unstable in standard PBS buffer (*Ls*LOx), or were not specific to L-lactate (*Pt*GOx). We therefore considered them unsuitable for application in L-lactate biosensors.

### Apparent Steady-State Kinetics

The Michaelis–Menten constants are important parameters when it comes to the selection and application of an enzyme in a biosensor. Here, we determined the apparent steady-state constants for L-lactate. The resulting Michaelis constants (*K*_m_) and catalytic constants (*k*_cat_) of the tested LOxs compare well with those of *Av*LOx (Table 2), mostly showing values in the same range. Only *As*LOx showed a *K*_m_ value noticeably higher than those of other LOxs, and *Sa*LOx and *Sd*LOx showed *k*_cat_ values lower than those for most others.

**Table 2:** Apparent Steady-State Kinetic Constants for the Oxidation of L-lactate by Different L-lactate Oxidases^a^

enzyme	*K*_m_[mM]	*k*_cat_[s^–1^]	*k*_cat_*/K*_m_[mM^–1^s^–1^]
*Sd*LOx	0.62 ± 0.07	14.06 ± 0.31	23
*Sa*LOx	0.95 ± 0.27	6.66 ± 0.46	7
*Cv*LOx	0.38 ± 0.04	41.42 ± 0.87	110
*Ehm*LOx	0.92 ± 0.18	52.04 ± 1.64	57
*Ci*LOx	0.58 ± 0.13	25.60 ± 0.87	44
*Mp*LOx	0.31 ± 0.10	51.26 ± 3.48	168
*As*LOx	2.11 ± 0.21	48.39 ± 1.36	23
*Av*LOx	0.33 ± 0.05	46.26 ± 1.20	140

a Reactions were performed in 11 mM PBS pH 7.4 at 30 °C and O_2_ as the electron acceptor. L-lactate concentrations varied from 0.05 to 50 mM.

### Pyruvate Inhibition

Product (pyruvate) inhibition is a known issue for LOx and can be especially problematic in the application on biosensors.^22,44^ We therefore assessed inhibition constants (*K*_i_) and types of inhibition for pyruvate by measuring steady-state kinetics in the presence of increasing concentrations of pyruvate (Table 3 and Figure S4). All enzymes showed mixed inhibition as the best-fit model when comparing competitive, uncompetitive, noncompetitive, and mixed models. Inhibition constants showed clear differences between the studied LOxs, with *Sa*LOx showing the lowest *K*_i_ values and hence the strongest inhibition, while *As*LOx and *Cv*LOx showing the highest *K*_i_ values, i.e., the least pronounced inhibition.

**Table 3:** Inhibition Constants for Pyruvate for Different L-Lactate Oxidases^a^

enzyme	*K*_*i*_[mM]	inhibition type
*Sd*LOx	2.04 ± 0.54	mixed
*Sa*LOx	0.86 ± 0.49	mixed
*Cv*LOx	12.78 ± 5.85	mixed
*Ehm*LOx	1.78 ± 1.91	mixed
*Ci*LOx	2.89 ± 1.41	mixed
*Mp*LOx	9.21 ± 1.67	mixed
*As*LOx	14.11 ± 2.42	mixed
*Av*LOx	7.94 ± 0.90	mixed

a Reactions were performed in 11 mM PBS pH 7.4 at 30 °C and O_2_ as the electron acceptor. L-lactate and pyruvate concentrations varied from 0.05 to 50 mM and 0.1 to 40 mM, respectively.

### pH Dependence of LOx Activity

Determination of pH optima was done in Britton-Robinson buffer (BRB) under standard assay conditions. The pH range where LOxs show 80–100% relative activity with L-lactate is 7.0–9.0 for *As*LOx, 7.5–9.5 for *Ci*LOx, 7.0–9.0 for *Cv*LOx, 7.0–8.5 for *Ehm*LOx, 7.0–8.5 for *Mp*LOx and, 9.5 for *Sa*LOx and *Sd*LOx (Figure S5). For comparison, *Av*LOx showed 80–100% activity in a pH range of 7.0–8.5, as previously reported.^26^ To determine if the use of BRB buffer affected enzyme activity, we compared BRB at pH 7.5 with standard PBS buffer at pH 7.4 (Figure S6). Overall, the measured activities were comparable, except for *As*LOx and *Sd*LOx, which showed ∼85% and ∼65% of activity in BRB, respectively, compared to PBS, and *Ehm*LOx, which showed ∼140% activity in BRB.

### Biosensor Matrix and Thermostability

To test the enzymes’ performance on a biosensor, the enzymes were immobilized on a rotating disc electrode (RDE) together with a recently reported matrix for electrochemical H_2_O_2_ detection, consisting of water-dispersible poly(3,4-ethylenedioxythiophene)-poly(styrenesulfonate) and PB (PEDOT:PSS–PB).^29^ This matrix has already been shown to work for L-lactate detection in exhaled breath condensate when immobilized together with LOx.^30^ The PEDOT:PSS matrix is a conductive, biocompatible polymer complex that can maintain the aqueous plasticity of the PEDGE-cross-linked enzyme by attracting water inside the matrix^45^ and has been widely used in the construction of conductive electrodes because of its stability, flexibility, and mechanical properties.^46^ Routine preparation of this biosensor matrix includes a 2-h drying step at 55 °C. Unfortunately, this step led to a strong loss of enzyme activity in our preparations. We, therefore, modified the conditions of the drying step to be performed at 4 °C in a desiccator instead. This completely abolished the losses of enzyme activity. The lower drying temperature seemed to impede the adsorption of the enzyme matrix on the electrode though, as we visually observed the polymer to detach more easily during electrode cleaning, compared to sensors prepared at 55 °C. The reason for the strong inactivation of the tested LOxs at 55 °C became evident when investigating the enzymes’ thermostabilities, determining T_50_ values, i.e., the temperature at which enzyme activity is reduced to 50% after 30 min of incubation (Table 4 and Figure S7). All novel LOxs show T_50_ values lower than that of *Av*LOx and some well below 55 °C.

**Table 4:** Thermostability of Various L-Lactate Oxidases^a^

	*Sd*LOx	*Sa*LOx	*Cv*LOx	*Ehm*LOx	*Ci*LOx	*Mp***LOx**	*As***LOx**	*Av***LOx**
T_50_ [°C]	42.5	36.6	38.2	54.0	48.6	37.4	42.2	54.9

a Residual enzyme activities were determined under standard assay conditions after incubation at various temperatures for 30 min. T_50_ values were calculated using an iterative sigmoidal fit of the mean of quadruplicate incubations.

### Rotating Disk Electrode Measurements

As the working electrodes of the biosensors, we used rotating disc electrodes (RDEs), allowing for measurements with different rotational speeds. Amperometry measurements of RDEs were recorded at room temperature at potentials that ensured mass transport-controlled currents, as determined for each immobilized LOx individually (Figure S8), ranging from −0.15 to +0.015 V (Table S3). The total recording time for each RDE lasted 45 s and included three different rotational speeds. Currents were recorded for a range of L-lactate and hydrogen peroxide concentrations, respectively. The recorded reciprocal currents plotted versus rotational speeds as angular velocity were implemented as a base for Koutecký–Levich (KL) analysis (Figure S9). The KL analysis made it possible to estimate currents independent of the mass transport in bulk solution by linearly extrapolating the recorded data to infinite rotational speed (KL intercept). The slopes of the respective linear fits (KL slopes) provided information about the diffusivity of L-lactate and hydrogen peroxide in the bulk solution. The resulting diffusion coefficients (D_lactate_ and ) for L-lactate and hydrogen peroxide (Table S4) were consistent with previously published results^29,30^ and confirmed that prepared biosensors were all functional and showed comparable responses between the respective LOx/PEDOT:PSS–PB matrixes.

**Figure 3:**
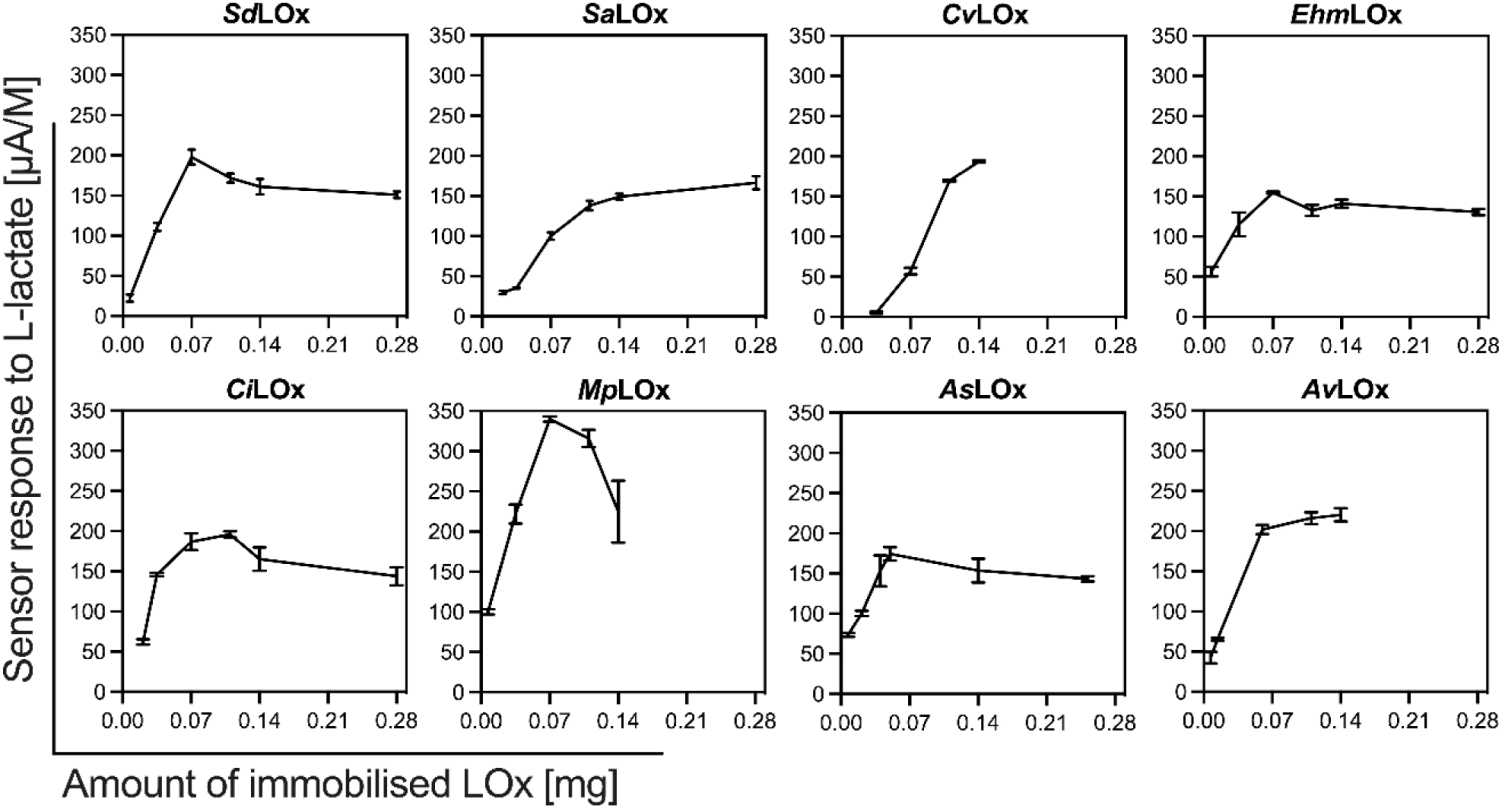
Sensor responses to L-lactate plotted against the amount of immobilized enzyme (assuming 100% efficiency of immobilization) on the sensor. Sensor responses are taken from the linear slopes of KL intercepts plotted versus their corresponding L-lactate concentrations of 50–250 μM (*n* = 3).

To identify the optimal amount of immobilized LOx for each biosensor, multiple sensors with increasing amounts of LOx were prepared and the corresponding sensor responses, given as current per L-lactate concentration, were determined (Figure 3). For low LOx concentrations, sensor responses quickly increased with the amount of immobilized enzyme. Typical for processes of enzyme immobilization though, at higher amounts of enzyme, the responses saturated at a maximum or decreased again when the amount of LOx was further increased. This resulted in different optimal quantities of enzyme for the constructed sensors, depending on the applied LOx, respectively.

### Sensor Catalytic Turnover Rates

**Figure 4:**
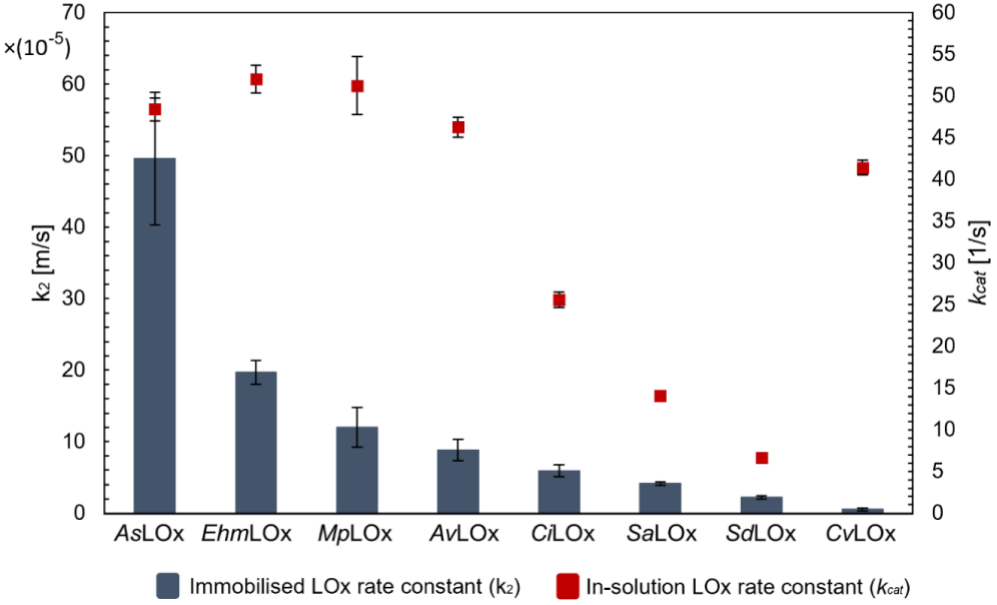
Comparison of apparent rate constants for LOxs immobilized on RDEs in a PEDOT:PSS–PB matrix (*k*_2_) with the respective apparent rate constants measured in solution (*k*_cat_). *k*_2_ values were estimated from the Koutecký–Levich analysis and describe the overall enzyme reaction rate on the sensor.

KL analysis further enabled us to estimate apparent rate constants for LOxs immobilized on RDEs. The resulting *k*_2_ values describe the overall enzymatic reaction that contributes to the generated sensor current. When comparing the respective *k*_2_ values for each tested LOx (blue columns in Figure 4), we found the immobilized enzymes *As*LOx, *Ehm*LOx and *Mp*LOx showing higher catalytic rate constants than *Av*LOx. Noteworthy, *As*LOx and *Ehm*LOx show *k*_2_ values more than five and two times higher than the *Av*LOx sensor, respectively. Finally, we compare the enzymatic rate constants determined for immobilized enzymes on the sensors (*k*_2_), with the enzymatic rate constants measured for each LOx in solution (*k*_cat_) and show that there is no direct correlation between these values (Figure 4).

Although a certain trend can be detected that the *k*_cat_ values and the sensor *k*_2_ correlate to some extent–enzymes with low *k*_cat_ values also show lower sensor *k*_2_ and vice versa, distinct deviations from this correlation from a *k*_cat_/*k*_2_ correlation are obvious as well–enzymes with comparable *k*_cat_ values of ∼40–50 s^–1^ show the highest (*As*LOx) as well as the lowest *k*_2_ values (*Cv*LOx) on the sensor. Apparently, not only the turnover rate of the enzyme in solution but also several additional factors determine the performance and suitability of an enzyme for a specific biosensor. Considering this, it becomes more evident that it is important to test multiple enzymes in the development of a biosensor.

## Discussion

Recently, we analyzed the α-hydroxy acid oxidoreductase superfamily using SSN, phylogeny, and sequence analysis. We showed that oxidoreductases specific for L-lactate group mainly in one distinct cluster in the SSN analysis, and that sequences exhibiting oxygen reactivity form a distinct subgroup (LOxs) within this cluster, separate from sequences with negligible oxygen reactivity (LDHs).^26^ Such LOxs are potential enzymes for the development of improved L-lactate biosensors. To date, we have reported only preliminary activity screening data of these novel LOxs. Here we report a more detailed biochemical and electrochemical characterization of seven selected LOxs, evaluating their suitability for L-lactate biosensor application. Initially we selected 11 unstudied LOx members, representing a broad sequence variation within the LOx clade, expressing them together with *Av*LOx in *E. coli* shaken flask cultures. Even though we obtained LOx activity for all expressions, the yields and activities varied strongly for the various enzymes. Expressibility of some of the genes was excellent, whereas expression of *Av*LOx, which is used as the golden standard in L-lactate biosensor development, along with few other selected *lox* genes, was repeatedly very poor. Since enzymes can be a relevant cost factor in the production of biosensors, their expression yields might determine the economic feasibility of a biosensor. The reason for our continuously very low expression yields of *Av*LOx is still unclear, especially since we used a pET-vector expression system, commonly used also by other groups.^34,39,47^ To still be able to compare our measured biochemical and electrochemical data with those of *Av*LOx, we substituted in-house produced *Av*LOx with the commercial product.

Except for *Pt*GlOx, all tested oxidases preferred L-lactate as the substrate and showed considerably higher activities in the presence of oxygen than with the alternative electron acceptors Fc^+^ and DCIP; hence, they can be considered true L-lactate oxidases. *Pt*GlOx, on the other hand, showed its highest activity with glycolate and, in general, exhibited a much broader reactivity, utilizing various electron donor substrates. This difference in substrate specificity could be caused by a 25 amino acid-long insertion at the active site lid-loop of *Pt*GlOx, which is known to be important for substrate recognition in *Av*LOx.^24^ Four enzymes (*Ed*LOx, *Cb*LOx, *Ls*LOx, and *Pt*GlOx) were considered unsuitable for biosensor application since they either showed problems during their production or did not prefer L-lactate as a substrate. The remaining seven enzymes were biochemically studied in more detail.

The characterized LOx enzymes all compared very well with respect to their catalytic properties (apparent *K*_m_ and *k*_cat_), which also showed a high resemblance to the properties of *Av*LOx. Product inhibition (*K*_*i*_) and thermostability (T_50_), on the other hand, varied substantially among the studied enzymes. Such, and other properties, should be taken into consideration when selecting a suitable enzyme for the construction of a biosensor. If a specific enzymatic property is compatible with a biosensor is not generalizable though. Some enzymatic properties can be influenced or compensated by sensor design, and if a property is critical to the sensor is ultimately dependent on the intended application. Thermostability, as well as turnover rates for example, can be influenced by the type of immobilization method used. The presence or absence of a membrane will influence the importance of the enzymatic *K*_m_ and *K*_i_ values. The type of sample or measurement condition might make it necessary for the enzyme to withstand harsh conditions, tolerate inhibitors or operate in a certain pH range. Also, the mode of operation, sensor production, transport, and storage conditions can have specific requirements that need to be fulfilled by the enzyme. Enzymatic properties should therefore be considered if deemed relevant for a specific sensor.

Since it is well-known that the properties of soluble enzymes may differ from those of the same enzyme when immobilized,^48,49^ we tested and compared the newly characterized LOxs as immobilized enzymes on RDEs, embedded in a PEDOT:PSS–PB matrix. Measurements of different amounts of LOx on the sensor showed that the tested enzymes have different optimal amounts of LOx with respect to sensor responses. By means of KL analysis, catalytic rate constants (*k*_2_) were estimated separately for the enzymes embedded in the sensor matrix. These apparent *k*_2_ values were further compared with the enzymes’ *k*_cat_ values measured in solution. Although these two rate constants cannot be compared directly, they might give a first picture of how the various enzymes perform in comparison under the two conditions. Our measurements showed that even enzymes with comparable *k*_cat_ values can show very diverse catalytic performances on the sensors. This indicates that an enzyme’s performance on a sensor is determined by several factors, and it is not trivial to predict its performance only from its properties in solution. For glucose oxidase for example, it was shown that the immobilization method has a relevant effect on its kinetic properties when immobilized on an electrode.^50,51^

For both, the free and the immobilized enzyme, we determined the *apparent* kinetic constants for L-lactate in the two-substrate reaction of LOx by keeping the concentration of one substrate (oxygen, concentration in ambient air) constant while varying the concentration of the second substrate, L-lactate. Determination of the *true* kinetic constants by applying various fixed concentrations of oxygen can give information on the reaction mechanism of LOxs, yet from an applied perspective, when the LOx-based sensors will be used with ambient oxygen and air, the apparent constants provide sufficient information.

## Conclusion

When constructing a biosensor, differing numbers of catalytic units of an immobilized enzyme may contribute to the sensors’ performance, depending on the enzyme, the sensor matrix, and the immobilization technique. This means that the final performance of a sensor with a specific enzyme is determined by a complex orchestration of multiple factors, including biophysical and biochemical properties of the enzyme, as well as the type of electrode, applied mediator, matrix composition, and immobilization technique. It therefore seems advantageous to test a variety of enzymes for a specific sensor type to find the best-performing enzyme under the applied sensor design. Unfortunately, to date, it is still uncommon for multiple enzymes to be tested and compared on one sensor design. Biosensor development commonly focuses more on sensor architecture and inorganic sensor components and typically leaves out the bioelement of the biosensor.

Our study shows that seven previously unexplored LOxs could be used as bioelements in L-lactate biosensors as alternatives to *Av*LOx. While some biochemical features, like catalytic constants and pH optima, hardly differed between the tested enzymes, other factors, like expression yields, pyruvate inhibition and thermostability, showed larger variation. In the end, when tested on electrodes, three LOx enzymes (*As*LOx, *Ehm*LOx, and *Mp*LOx) showed higher catalytic rate constants than the commercial enzyme *Av*LOx, making them interesting candidates to be applied in future studies on L-lactate biosensors. With our results, we want to highlight that each biosensor represents a unique combination of electrode and enzyme, which has distinct properties and limitations that cannot be easily predicted from biochemical enzyme data. Potentially better performing biosensors can therefore easily be overlooked when focusing only on a single biocatalyst in sensor development.

## Materials and Methods

### Chemicals

All buffer components were purchased from Sigma-Aldrich. All aqueous solutions were prepared with deionized water. Phosphate-buffered saline (PBS) 11 mM, pH 7.4, with 137 mM NaCl and 3 mM KCl was used as a standard buffer for all experiments unless stated otherwise. Ferrocenium hexafluorophosphate (FcPF_6_) was purchased from Santa Cruz Biotechnology (USA); 10-acetyl-3,7-dihydroxyphenoxazine (AmplexRed) from Chemodex (Switzerland); horseradish peroxidase (HRP), 2,6-dichlorophenol-indophenol sodium salt hydrate (DCIP), sodium L-lactate, and sodium glycolate were from Sigma-Aldrich; L-2-hydroxyvaleric acid (S-2-hydroxyvaleric acid) was from BLD Pharmatech (China); L-2-hydroxy-n-octanoic acid from TCI (Japan); S-(+)-mandelic acid from Fluorochem (UK). Poly(3,4-ethylenedioxythiophene) polystyrenesulfonate (PEDOT:PSS), 1.3% dispersed in water; glycerol >99%; poly(ethylene glycol) diglycidyl ether (PEGDE) were all purchased from Sigma-Aldrich (Germany) and potassium ferrocyanide trihydrate (Prussian Blue) supplied by Fisher Chemical.

### Sequence Alignment and Phylogenetic Analysis

The 12 α-hydroxy acid oxidoreductases listed in Table S1 were aligned by MAFFT v7.402 (algorithm G-INS-i)^52^ and the best amino acid substitution model for tree inference according to AIC was determined to be LG+I+G4 by ModelTest-NG.^53^ Maximum likelihood phylogenetic tree inference was conducted using default settings from RAxML-NG,^54^ and the resulting tree was visualized in MEGA 7^55^ and rooted on the midpoint.

### *Lox* Gene Expression

The codon-optimized *lox* genes for *E. coli* expression in a pET-21(+) vector were purchased from Twist Bioscience (San Francisco, USA). The genes were modified with an N-terminal purification tag, as reported previously.^26^ Plasmids were transformed into *E. coli* BL21 (DE3) and sequences of single colonies were confirmed by Sanger sequencing (Microsynth, Austria). Expression of recombinant genes in *E. coli* was done similarly to previous reports^26^ using LB or TB medium in shaken flask cultures (media components purchased from Carl Roth, Germany). Protein expression was induced by the addition of 250 μM β-D-1-thiogalactopyranoside (Sigma-Aldrich, Germany). After 19 h, cells were harvested and washed once, and pellets were stored at −20 °C.

### Protein Purification

As previously reported,^26^ cells were disrupted using multiple cycles in a French Press, and after centrifugation, crude extracts were filtered through a 0.22-μm membrane filter. A kta FPLC system (GE Healthcare, USA) was used to conduct IMAC purification on 5 mL HisTrap FF columns (Cytiva, USA). After binding the His-tagged proteins, the column was washed and loaded with HRV 3C protease (∼0.2 mg/mL of column volume) and left for 19 h of incubation at 4 °C. After cleavage of the His-tags, proteins were eluted with 50 mM PPB, pH 6.5 in a single fraction, concentrated in Amicon centrifugal filters (MWCO 10 kDa, Merck), rebuffered to 11 mM PBS, pH 7.4, and stored at 4 °C. Purity of the enzymes was evaluated by SDS-PAGE analysis, performed with commercial precast 4–20% Mini-PROTEAN TGX Stain-Free Protein gels. Band intensities were analyzed by ImageLab 6.1 (Bio-Rad). Protein concentrations of pure enzymes were calculated according to their absorbance at 280 nm using theoretical extinction coefficients as determined from amino acid sequences by the ExPASy tool ProtParam.^56^

### Enzyme Activity Assays

Enzymatic activities were recorded in 300 μL total volumes in flat bottom microtiter plates using an EnSpire multimode plate reader with temperature regulation (PerkinElmer, USA). Oxidase activity was determined by the standard AmplexRed assay; a reaction coupled with 7.1 U/mL horseradish peroxidase (181 U/mg) containing 0.05 mM AmplexRed (resorufin: ε560 nm = 54.0 mM^–1^ cm^–1^) under ambient oxygen concentrations of ∼250 μM at 30 °C. Dehydrogenase activities were measured as reported before, using direct dye-mediated assays containing either 300 μM DCIP or 160 μM FcPF_6_.^26^ All activity assays contained the electron donor substrates sodium L-lactate, sodium glycolate, L-2-hydroxyvaleric acid, L-2-hydroxy-n-octanoic acid, or L-mandelic acid at 10 mM dissolved in PBS. Using the AmplexRed (oxygen) assay, apparent steady-state kinetic constants were determined using L-lactate concentrations ranging from 0.05 to 50 mM and fitting the data with the Michaelis–Menten equation (v = (*v*_max_*[S]/(*K*_m_+[S])) using GraphPad Prism Version 9.5.1 for iOS (GraphPad Software, USA, www.graphpad.com). Turnover rates were calculated based on monomeric enzyme masses. One unit of enzyme was defined as the amount that oxidizes 1 μmol of L-lactate per minute at 30 °C. Inhibition constants were determined by measuring Michaelis–Menten kinetics (0.05–100 mM L-lactate) at three individual concentrations of pyruvate (from 0.1 to 40 mM) for each enzyme, respectively. Recorded data were fitted with four inhibition models (competitive, uncompetitive, noncompetitive, and mixed), compared, and selected using the extra sum-of-squares F-test, available in GraphPad Prism. The effect of pH on LOx activity was evaluated using the AmplexRed assay in the presence of oxygen in 40 mM Britton-Robinson universal buffer (BRB), containing 40 mM phosphoric, boric, and acetic acid, adjusted with 4 M KOH to a pH varying from 4.5 to 9.5. The thermal inactivation of LOxs was determined after the incubation of enzyme samples in quadruplicates with a fixed protein concentration of ∼10 mg/mL at various temperatures for 30 min. Residual activities were determined using a standard AmplexRed assay, and resulting data were fitted iteratively with a sigmoidal function using the Microsoft Excel plugin Solver and least-squares regression.

### Protein Immobilization on RDE

Aliquots (500 μL) of purified LOxs (2 mg/mL) were concentrated with a rotational vacuum concentrator (RVC 2–18 CDPLUS, Martin Christ, Germany) by applying ∼10 mbar of pressure for 2 h. The highly viscous protein sample was transferred to a vacuum desiccator and left overnight (∼19 h) at 4 °C for complete water removal. Thus, prepared enzyme powder was stored at −20 °C until further use. The enzyme immobilization procedure for rotational disk electrodes was adapted from previous publications, and the PEDOT:PSS–PB solution was prepared according to published protocols.^29,30^ Briefly, the concentration of PB was ∼43 mM, PEDOT—0.5 wt %, and PSS ∼ 43 mM. Dried LOx samples were dissolved in 100 mM PPB, pH 7.4, containing 100 mM KCl, and bovine serum albumin (BSA) was added to enhance the stability of LOx. The amount of BSA was adjusted to the applied amounts of LOx (0.007, 0.014, 0.021, 0.035, 0.040, 0.050, 0.060, 0.070, 0.112, 0.140, 0.252, or 0.280 mg) respectively to maintain a ratio of LOx to BSA of 3 to 2. The total amount of protein immobilized on the RDE never exceeded 0.5 mg. PEGDE (>1%) was used as a cross-linking agent. The protein solution, composed of LOx and BSA, was mixed with PEGDE and PEDOT:PSS–PB solution in a ratio of 1:2.5, drop-casted onto the RDE and dried in a silica gel desiccant container for >4 h, at 4 °C.

### RDE Measurements

Electrochemical measurements of immobilized LOx were done in 20 mL of 100 mM PPB, pH 7.4, containing 100 mM KCl and recorded at room temperature using the potentiostat CompactStat (Ivium Technologies, The Netherlands). A glassy carbon rotational disk electrode (Pine Research Instrumentation, USA) with a diameter of 5 mm was used as a working electrode in a three-electrode electrochemical cell. The RDE was mounted on a Pine Research MSR Rotator. All measurements were recorded versus an aqueous Ag/AgCl reference electrode and a platinum auxiliary electrode. Since no extreme current values were present in the recorded amperograms (not shown), the Savitzky–Golay Filter (available in GraphPad Prism) was used to smooth the data. The applied potentials for the respective enzymes were deducted from cyclic voltammograms for oxidized and reduced PB, to ensure that currents were mass transport-controlled and PB was in a fully reduced state. Recording of steady-state currents was performed at rotational speeds of 49, 100, and 169 rpm. These rotational speeds were selected to be high enough to strongly reduce the natural diffusion layer thickness of ∼0.5 mm^57^ but at the same time keep well below rates that would result in turbulent flow. Measurements were performed at 6 concentrations of L-lactate (50–250 μM) and hydrogen peroxide (50–175 μM), respectively. Concentration of the hydrogen peroxide stock solution was determined using potassium permanganate titration, and dilutions were prepared from a 30% w/v stock solution (Fisher Chemical, USA).

### Koutecký–Levich Analysis

The expression of the Koutecký–Levich (KL) equation was analyzed by plotting the reciprocal current () against the reciprocal square root of the angular velocity () from RDE measurements. The RDE’s resistance factors and the Prussian Blue–Prussian White transformation rate were assumed to be the same as described and discussed before,^29,30^ since the same sensor architecture was used in the current study. The overall current recorded with the applied RDE method is presented as the reciprocal sum of resistances by the KL eq (eq 1)
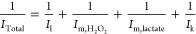
1where  is the mass transport in bulk solution,  is the transport of H_2_O_2_ in the LOx/PEDOT:PSS–PB matrix,  is the transport of L-lactate in the LOx/PEDOT:PSS–PB matrix, and  is the enzyme reaction. Diffusion coefficients of L-lactate and hydrogen peroxide were calculated from measurements at different L-lactate and hydrogen peroxide concentrations, respectively, using the corresponding slope of the KL plot () and eq 2,
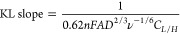
2where *n* is the number of moles of transferred electrons, *F* is the Faraday constant, *A* is the RDE surface area, *D* is the diffusion coefficient of the analyte, ν is the kinematic viscosity and *C*_*L/H*_ is the respective concentration of L-lactate or hydrogen peroxide. The presented diffusion coefficients are mean values over all of the measured concentrations.

By using the reciprocal currents of extrapolated data to infinite angular velocity (*KL intercepts*), the currents become independent of mass transport in bulk solution and the KL equation reduces to (). Since, furthermore, the current is mainly limited by the enzyme reaction at low concentrations of LOx, we estimated the sensor catalytic rate constants (*k*_2_) for all tested enzymes at their lowest applied concentration, from their KL intercepts (), using eq 3 and *K*_m_ values as determined in solution.
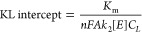
3

Resulting *k*_2_ values are considered heterogeneous rate constants as they describe the overall enzymatic reaction including oxidation of L-lactate and reduction of oxygen. Since the measured electric current results from an electrode surface but is dependent on the volumetric concentration of the enzyme, *k*_2_ is resulting in m/s. Presented *k*_2_ values are mean values of *k*_2_ calculated for all measured L-lactate concentrations.

To determine the optimal amounts of LOx being immobilized on the sensors, the *KL intercepts* were plotted versus their respective L-lactate concentrations for each amount of immobilized LOx tested. Slopes of the linear range of these plots, describing the sensor response to L-lactate (μA/M), were then plotted versus the amount of immobilized LOx. The amount of LOx giving the maximum sensor response to L-lactate was considered optimal.

## Supporting Information Available

Table of protein sequence IDs, sequence alignment overview, purification table, UV–vis spectra, substrate (electron donors’) structures, effects of pyruvate inhibition on LOx activities, pH profiles, buffer comparisons, thermal inactivation curves, cyclic voltammogram recordings and table of applied potentials in chronoamperometry (PDF)

## Abbreviations

HAOx: α-hydroxy acid oxidoreductase
LOx: L-lactate oxidase
BRB: Britton–Robinson buffer
DCIP: 2,6-dichlorophenol-indophenol
Fc^+^: ferrocenium ion
FcPF_6_: ferrocenium hexafluorophosphate
PBS: phosphate-buffered saline
HPLC: high-performance liquid chromatography
LC-MS: liquid chromatography–mass spectrometry
PPB: potassium phosphate buffer
FMNH_2_: reduced flavin mononucleotide
FMN: flavin mononucleotide
BSA: bovine serum albumin
OH^–^: hydroxide ion
RDE: rotating disc electrode
PB: Prussian Blue (i.e., potassium ferrocyanide trihydrate)
WE: working electrode
PEDOT: PSS, poly(3,4-ethylenedioxythiophene) polystyrenesulfonate
PEGDE: polyethylene glycol diglycidyl ether
HRV 3C: human rhinovirus protease 3C
SDS-PAGE: sodium dodecyl sulfate-polyacrylamide gel electrophoresis
SSN: sequence similarity network
IMAC: immobilized-metal affinity chromatography
UV–vis: ultraviolet–visible spectroscopy
