# Supplementary material for: More Than One Enzyme: Exploring Alternative FMN-Dependent L-Lactate Oxidases for Biosensor Development
Source: ACS Omega. 2024 Jun 26;9(27):29442–52. doi: 10.1021/acsomega.4c01897 (PMC11238220; doi:10.1021/acsomega.4c01897)
Supplement: Supplementary file 1 — ao4c01897_si_001.pdf [file ao4c01897_si_001.pdf]

Supporting Information

More Than One Enzyme: Exploring Alternative FMN-dependent L-lactate Oxidases for Biosensor Development

Lidiia Tsvik[1,2], Shulin Zhang[3], Danny O'Hare[3], Dietmar Haltrich[1] and Leander Sützl[1*]

[1] Laboratory of Food Biotechnology, Department of Food Science and Technology, University of Natural Resources and Life Sciences, Vienna, Muthgasse 11, A-1190 Wien, Austria

[2] Doctoral Programme 'Biomolecular Technology of Proteins (BioToP)', University of Natural Resources and Life Sciences, Vienna, Muthgasse 18, A-1190 Wien, Austria

[3] Department of Bioengineering, Imperial College London, London SW72AZ, U.K.

Table S1: List of eleven LOx sequences and *Av*LOx that were studied in this work. Sequence IDs correspond either to UniProtKB or NCBI database entries.

Enzyme	Species	Sequence ID
*Av*LOx	*Aerococcus viridans*	Q44467
*Sd*LOx	*Streptococcus dysgalactiae subsp. equisimilis*	A0A1C2C8T5
*Ed*LOx	*Enterococcus durans*	A0A367CFL5
*Ls*LOx	*Lactobacillus sakei subsp. sakei*	A0A4R5MXH1
*Sa*LOx	*Streptococcus anginosus*	A0A0P0N9S2
*Cv*LOx	*Carnobacterium viridans*	A0A1H0ZFB3
*Ehm*LOx	*Enterococcus hermanniensis*	A0A1L8TI44
*Ci*LOx	*Carnobacterium inhibens subsp. gilichinskyi*	U5S7L3
*Mp*LOx	*Marinilactibacillus piezotolerans*	A0A1I3VLW0
*As*LOx	*Aerococcus sanguinicola*	A0A109RDN9
*Pt*GlOx	*Pyrenophora triticirepentis*	A0A2W1FRD3
*Cb*LOx	*Calothrix brevissima*	WP_096646908.1

Figure S1: Sequence alignment of the twelve studied LOx sequences. The active site lid-loop residues at the alignment position 216–276 (amino acids 190–225 in *Av*LOx) are indicated by red bars. The figure was created using the alignment viewer MView from EMBL-EBI.

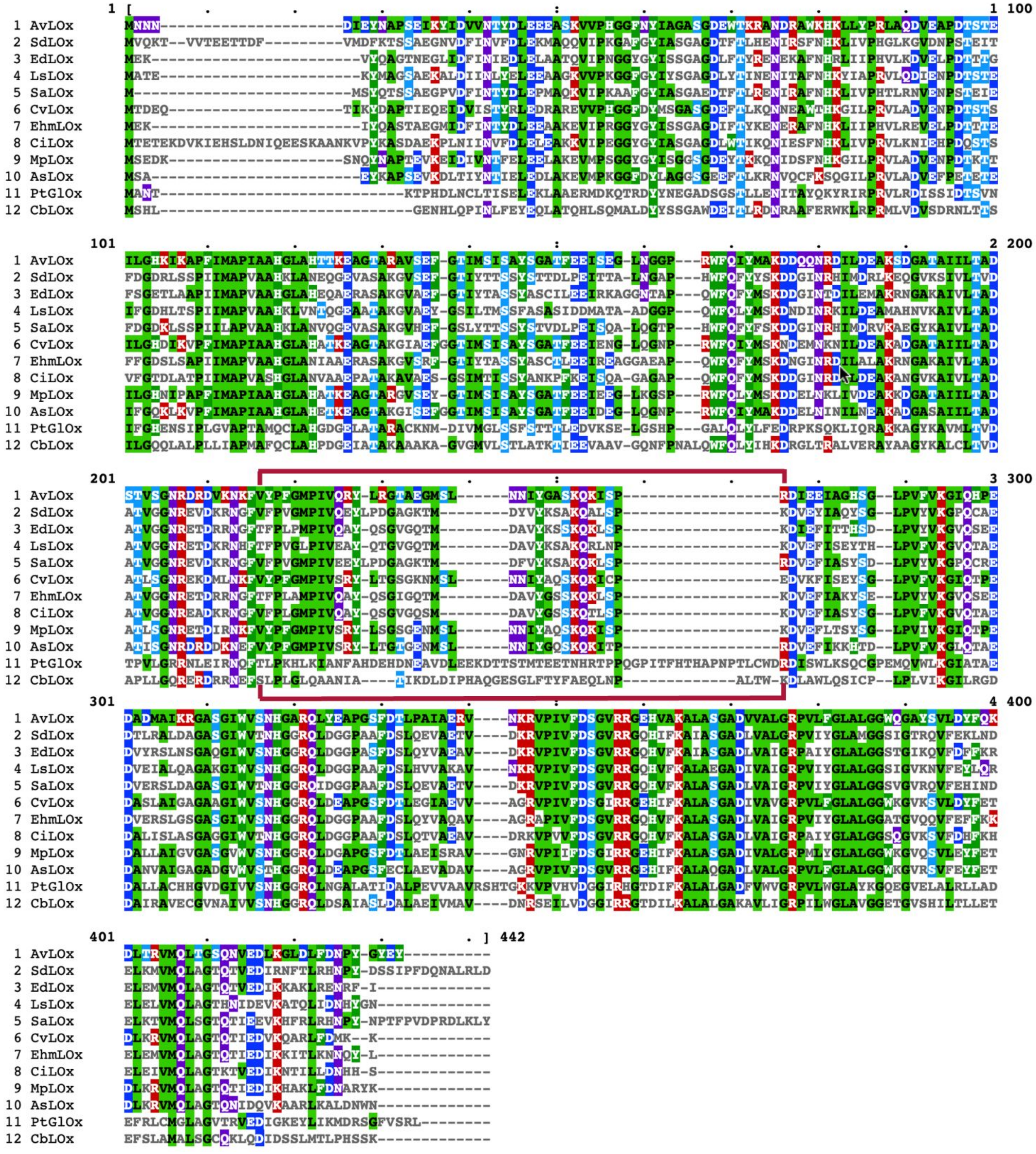

Table S2: Purification table of LOx enzymes expressed in this work.

**Enzyme**	**Cell pellet [g]**	**Purity by SDS PAGE [%]**	**Purified enzyme [mg]**	**Enzyme per cell pellet [mg/g]**
*Av*LOx	14	3	2	0.1
*Sd*LOx	19	97	6	0.4
*Ed*LOx	16	88	58	3.0
*Ls*LOx	35	98	134	3.8
*Sa*LOx	17	100	124	7.4
*Cv*LOx	20	99	30	1.5
*Ehm*LOx	15	99	67	4.5
*Ci*LOx	17	99	48	2.9
*Mp*LOx	14	92	91	6.3
*As*LOx	16	94	280	17.7
*Pt*GlOx	16	53	8	0.5
*Cb*LOx	13	10	3	0.2

Figure S2: UV-vis absorption spectra of the oxidised (black line) and reduced form (dashed grey line) of purified L-lactate oxidases. The two absorption maxima of the oxidised FMN cofactor are indicated in each spectrum. Spectra from *Cb*LOx and *Av*LOx could not be recorded due to insufficient amounts of pure enzyme.

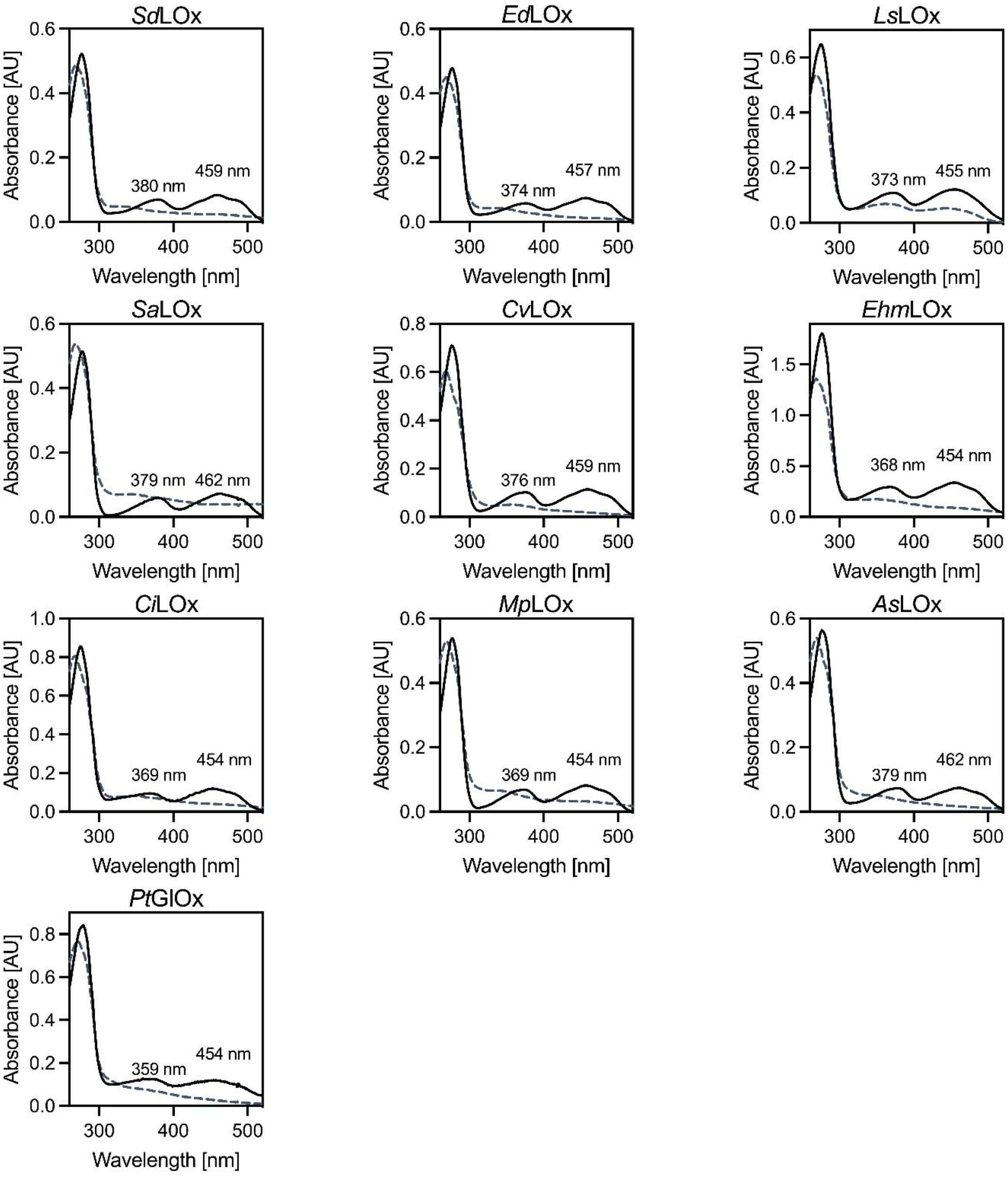

Figure S3: Structural comparison of the five different α-hydroxy acids used as electron donors for activity measurements. Chemical structures were created using ChemDraw version 22.2.0 by CambridgeSoft, based on PubChem structure coordinates.

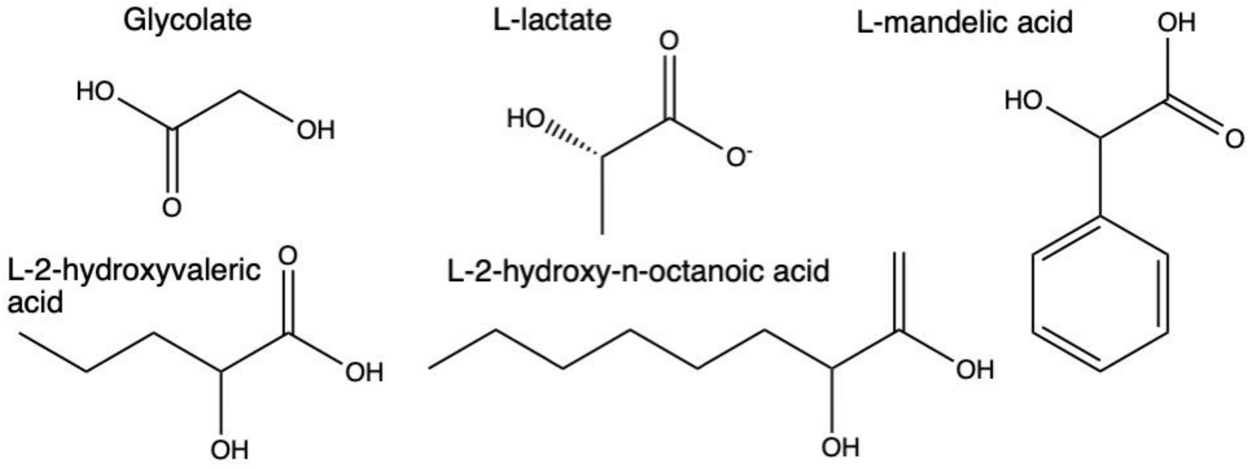

Figure S4: Effects of pyruvate inhibition on the L-lactate oxidase activity of different LOxs. The LOx activities were measured in dependence on varying L-lactate concentrations (0.05 – 100 mM), with 3 fixed pyruvate concentrations (0.1 – 40 mM), selected individually for every LOx. Measurement data is presented as an average of 4 measurements (for small standard deviations error bars are not shown) and fitted with Michaelis-Menten curves.

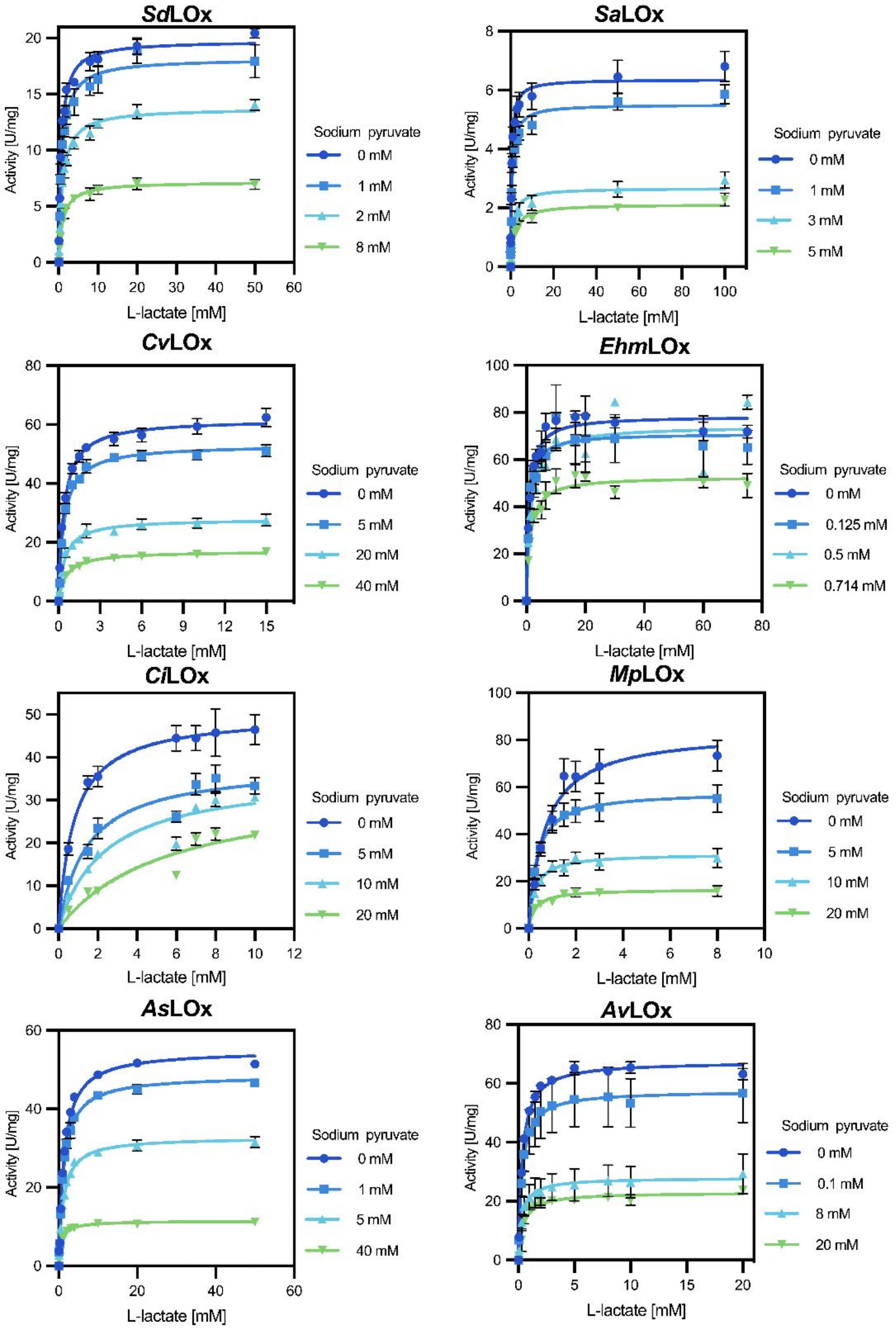

Figure S5: pH-dependent relative activities of different L-lactate oxidases measured with 10 mM L-lactate and oxygen as electron acceptor. Measurements were done in quadruplicates in 40 mM Britton Robinson universal buffer (BRB) pH 4.5–9.5. Activities are given relative to the highest recorded activity.

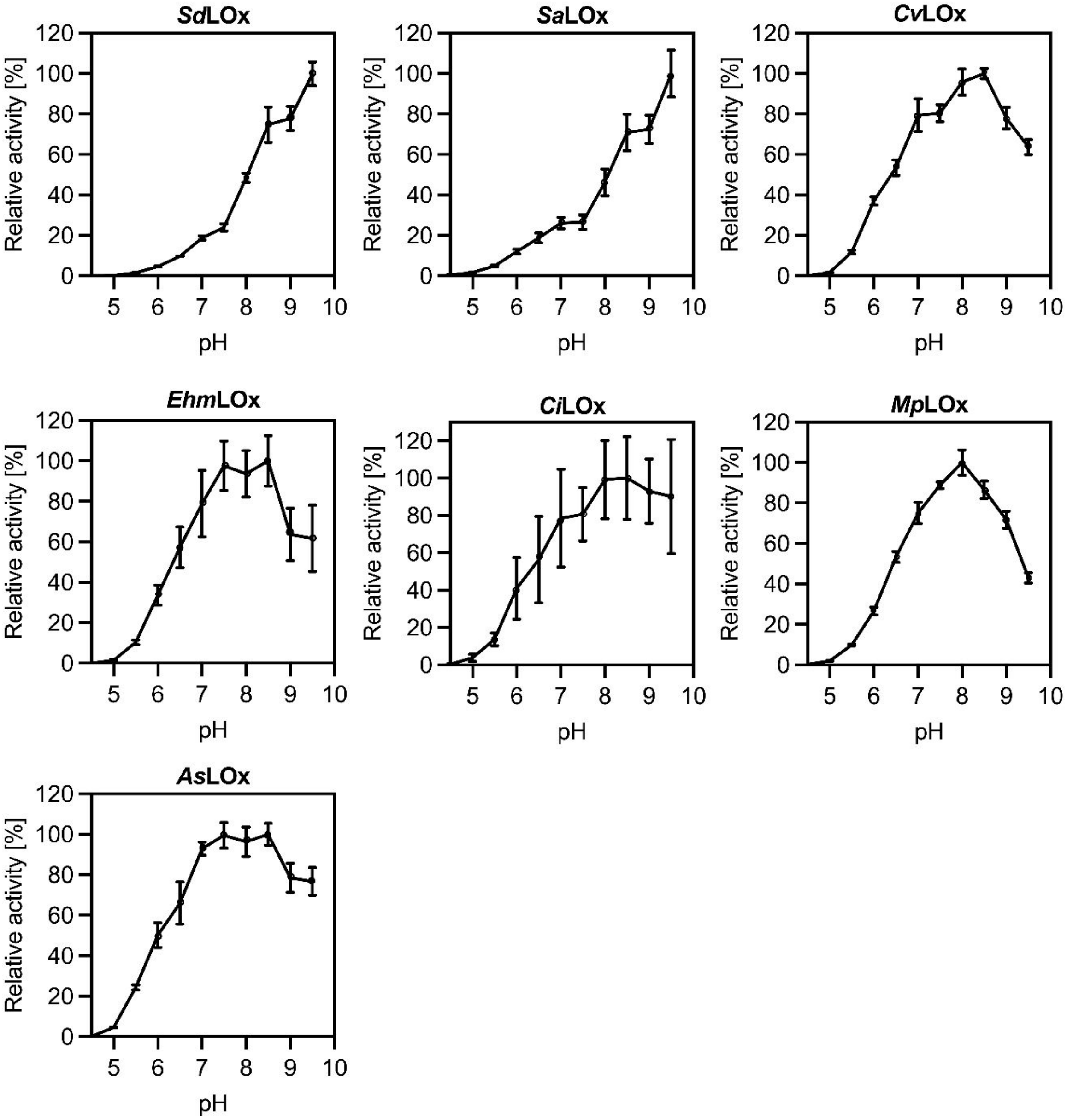

Figure S6: Relative comparison of L-lactate oxidase activity in two different buffer systems. Measurements were done in quadruplicates in 40 mM BRB pH 7.5 or 11 mM PBS pH 7.4 at 30°C using standard AmplexRed assay. Activities are given relative to the activity in PBS.

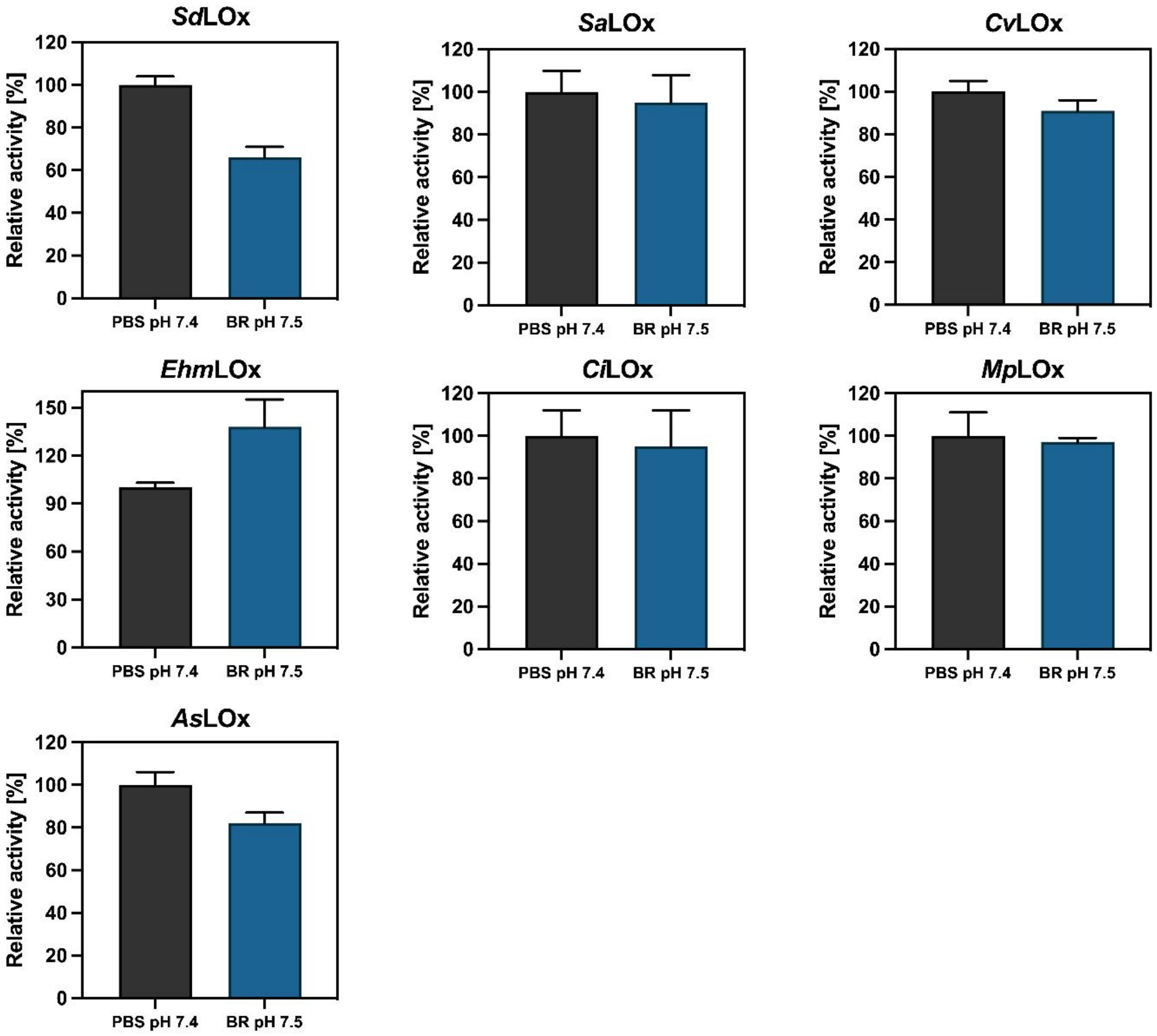

Figure S7: Sigmoidal fits of thermal inactivation data of L-lactate oxidases. Resulting T_{50} values are indicated by a red dot. Residual activities were measured in 11 mM PBS, pH 7.4, using the AmplexRed assay and 10 mM L-lactate. Values measured at 4°C were omitted from the sigmoidal fit for *Sa*LOx, *Ehm*LOx, *Ci*LOx and *As*LOx, respectively, due to an initial increase in activity upon incubation.

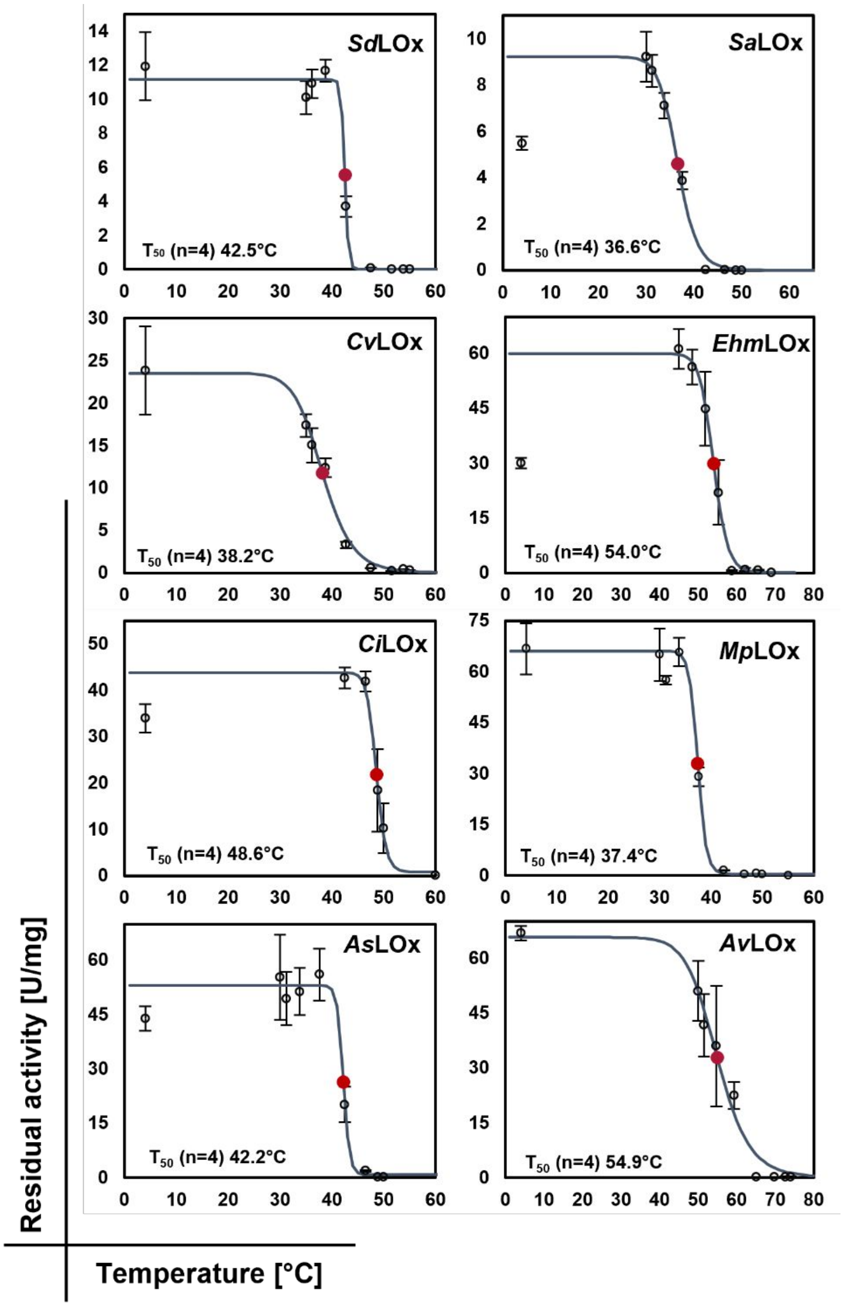

Figure S8: Cyclic voltammogram recordings of reduction and oxidation peaks of Prussian Blue immobilised with different L-lactate oxidases in PEDOT:PSS-PB.

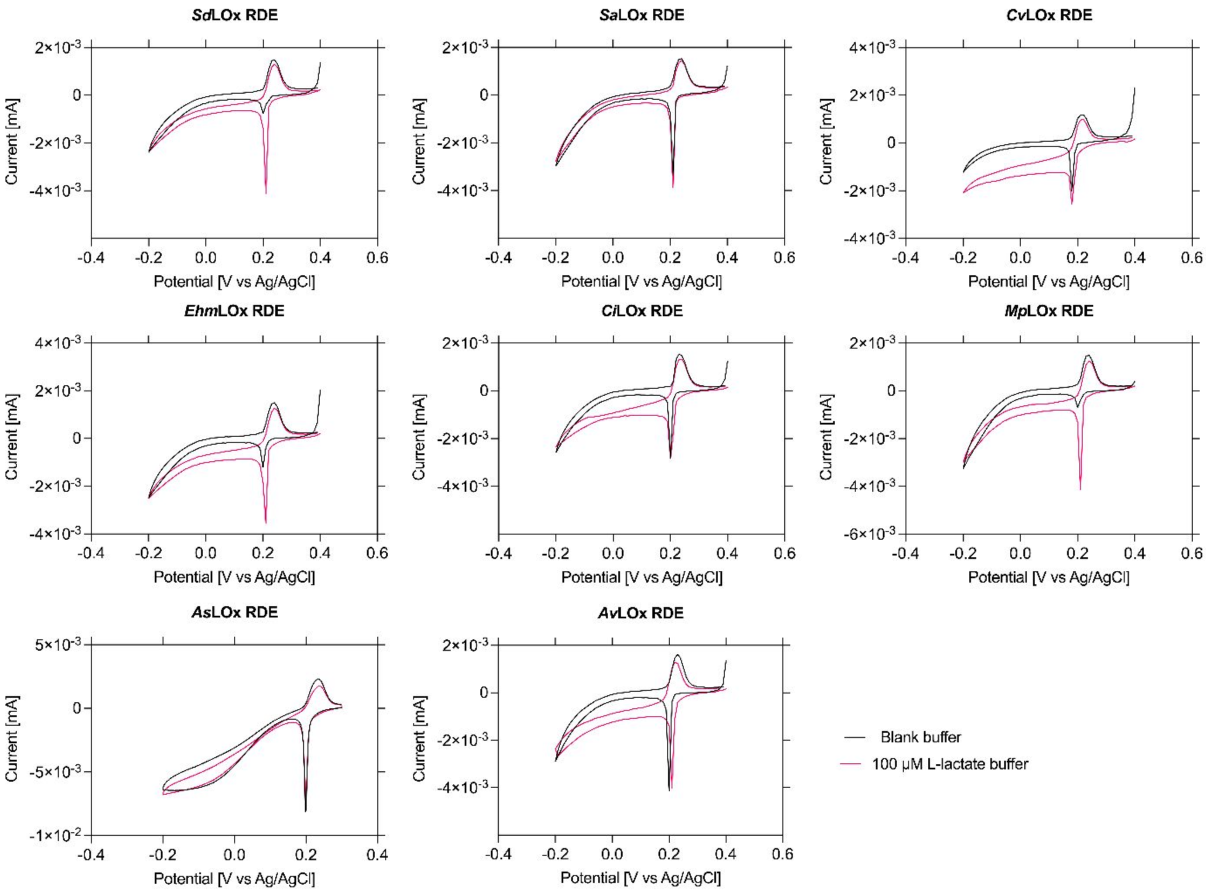

Table S3: Applied potentials in chronoamperometry for RDE prepared with different L-lactate oxidases in PEDOT:PSS-PB.

**Immobilised enzyme**	**Potential [V]**
*Sd*LOx	+0.015
*Sa*LOx	-0.010
*Cv*LOx	-0.100
*Ehm*LOx	+0.010
*Ci*LOx	-0.100
*Mp*LOx	-0.010
*As*LOx	-0.150
*Av*LOx	-0.100

Figure S9: Koutecký–Levich plots of the lowest applied amounts of LOx co-immobilised with PB on RDEs. The reciprocal currents recorded from 6 different L-lactate concentrations are plotted versus the reciprocal square root of the rotational speeds (49, 100 and 169 rpm), represented here as angular velocity. The current recorded for 0.05 mM L-lactate of *Cv*LOx *Sd*LOx, *Sa*LOx, and *Ehm*LOx did not give a stable signal and was therefore excluded from KL plots and further calculations. Displayed data represents mean values of 3 independent electrode measurements (for small standard deviations error bars are not shown).

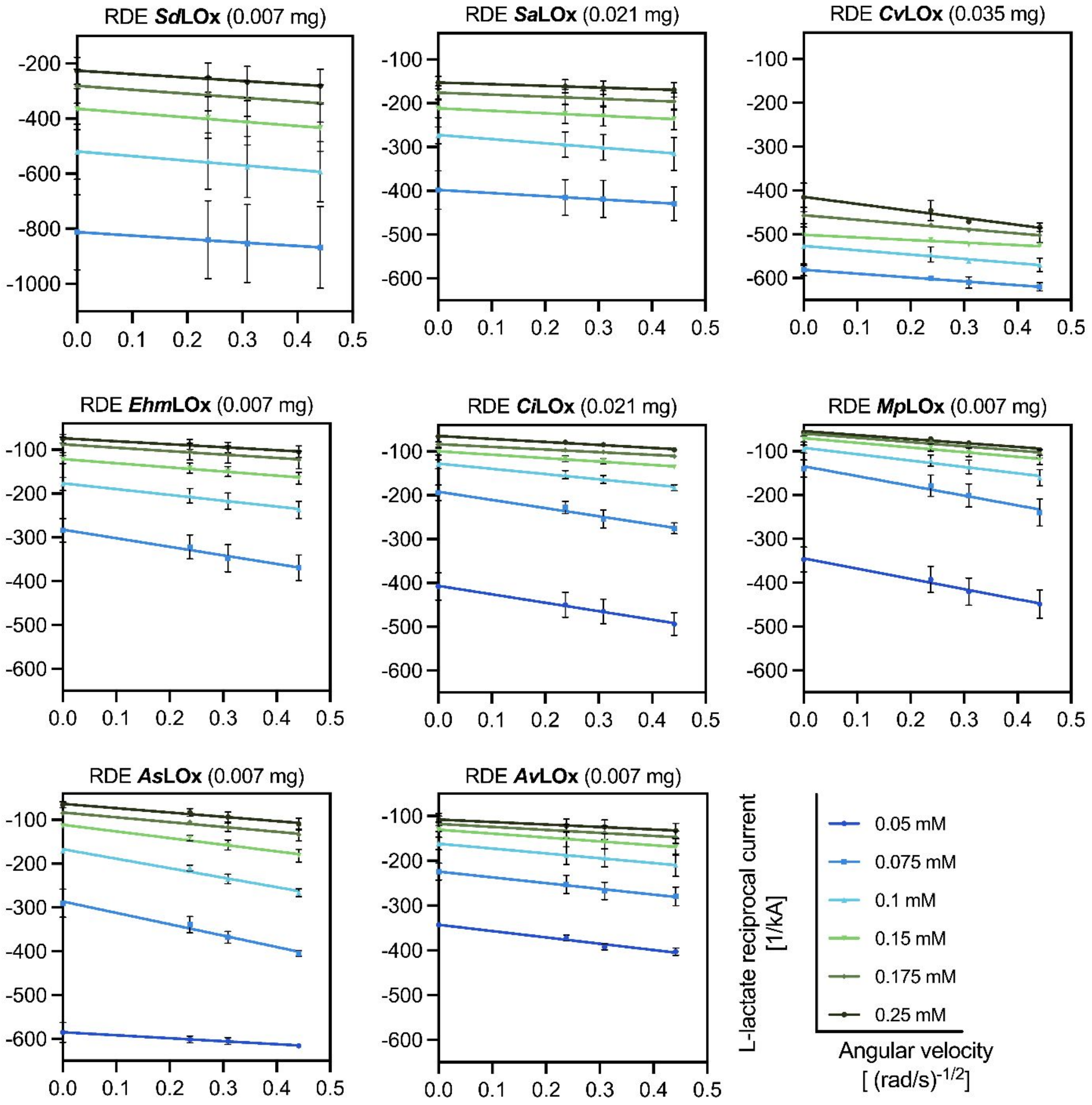

Table S4: Diffusion coefficients of L-lactate and hydrogen peroxide in bulk solution.

**LOx/PEDOT:PSS-PB**	**$D_{lactate}$ [m² s⁻¹]**	**D_{H2O2} [m² s⁻¹]**
*Sd*LOx	$(6.2 \pm 2.1) \cdot 10^{-10}$	$(2.3 \pm 0.6) \cdot 10^{-9}$
*Sa*LOx	$(7.5 \pm 2.0) \cdot 10^{-10}$	$(3.0 \pm 0.4) \cdot 10^{-9}$
*Cv*LOx	$(6.1 \pm 0.4) \cdot 10^{-10}$	$(1.9 \pm 0.8) \cdot 10^{-9}$
*Ehm*LOx	$(6.6 \pm 1.2) \cdot 10^{-10}$	$(1.5 \pm 0.4) \cdot 10^{-9}$
*Ci*LOx	$(6.7 \pm 0.2 \cdot 10^{-10}$	$(1.5 \pm 0.2) \cdot 10^{-9}$
*Mp*LOx	$(7.5 \pm 1.8) \cdot 10^{-10}$	$(2.2 \pm 0.7) \cdot 10^{-9}$
*As*LOx	$(6.1 \pm 0.4) \cdot 10^{-10}$	$(2.6 \pm 0.3) \cdot 10^{-9}$
*Av*LOx	$(7.8 \pm 1.5) \cdot 10^{-10}$	$(2.3 \pm 0.1) \cdot 10^{-9}$
